# Long-term evaluation of periprosthetic bone changes in ultra-short versus conventional stems in total hip arthroplasty: a 10-year follow-up of a randomised controlled trial

**DOI:** 10.1177/11207000251371283

**Published:** 2025-12-18

**Authors:** Michael Axenhus, Mats Salemyr, Sebastian Mukka, Martin Magnèli, Olof Sköldenberg

**Affiliations:** 1 Division of Orthopaedics, Department of Clinical Sciences at Danderyd Hospital, Karolinska Institute, Stockholm, Sweden; 2 Department of Surgical and Perioperative Sciences (Orthopaedics), Umeå University, Umeå, Sweden

**Keywords:** Conventional stem, femoral stem, long-term follow-up, periprosthetic bone mineral density, THA, ultra-short stem

## Abstract

**Background:**

Short stems in total hip arthroplasty (THA) have the potential to improve periprosthetic bone preservation compared to conventional stems due to a more anatomical distribution of biomechanical force. This study is a follow-up study of a randomised controlled trial using ultra-short and conventional stems in THA with the aim to investigate the long-term changes in periprosthetic bone mineral density (BMD) at 6 and 10 years post-THA.

**Methods:**

A cohort of 51 patients with hip osteoarthritis were randomized to either an ultra-short stem (*n* = 26) or a conventional stem (*n* = 25) group. Periprosthetic BMD was measured at 6- and 10-years post-surgery. Primary endpoint was BMD changes in Gruen zones 1 and 7. Lumbar spine, L1-4, BMD was used as an indicator of overall bone loss. Clinical outcome scores and BMD changes in Gruen zone 1-7 were used as secondary endpoints.

**Results:**

37 hips, 17 ultra-short stems and 20 conventional stems, were followed up until 10 years. At 6- and 10-years post-THA, the ultra-short stem group had less periprosthetic BMD reduction compared to the conventional stem group in Gruen zone 1; mean differences (%) were -17.6 (CI, -23.4–-10.6) and -18.3 (CI, -28.0–-9.0), respectively (*p* < 0.001). There was similar BMD loss in Gruen zone 7 and zones 1–7 between groups. Compared to overall bone loss, the ultra-short stem group lost less BMD than the conventional group. Adverse events and clinical outcomes did not differ between groups.

**Conclusions:**

Over a 10-year follow-up, THA using an ultra-short stem exhibited significantly reduced periprosthetic BMD loss in Gruen zone 1 compared to the conventional stem but this did not result in better clinical results. The observed preservation of bone density suggests potential long-term advantages of the ultra-short stem in minimising stress shielding and maintaining periprosthetic bone quality.

**Trial registration:**

ClinicalTrials.gov registration (number NCT01319227)

## Introduction

Total hip arthroplasty (THA) has traditionally used long stems which distribute biomechanical force down through the femur rather than the proximal part of the bone. Proximally porous and tapered titanium stems have been used in THA for decades and have good clinical outcomes.^1–3^ However, while providing stability and structural integrity, these stems have been shown to cause loss in bone mineral density (BMD) at the proximal femur. Periprosthetic bone remodeling and changes in BMD are integral features that influence long-term performance of the implant.^4^ Previous investigations have highlighted the significance of periprosthetic bone remodeling, particularly in minimizing stress shielding and reducing the risk of late periprosthetic fractures.^5,6^

Ultra-short stems emerged during the early 2010 as an alternative to conventional stems and offer theoretical benefits in the form of better anatomical force distribution and lower levels of BMD loss at the proximal femur, possibly mitigating the risk of periprosthetic fractures.^7,8^ Although these ultra-short stems have historically been associated with high failure rates, recent studies suggest that ultra-short stems have improved clinical and bone preservation outcomes when used appropriately.^9–11^ We previously performed a randomised controlled trial with a 24-month follow-up that revealed lower periprosthetic bone loss with the ultra-short stem compared to the conventional tapered stem.^12^

The aim of this study is to follow up our previous randomised controlled trial on ultra-short versus traditional stems and study their effect on periprosthetic BMD after 6 and 10 years. Our hypothesis is that ultra short stems can provide better preservation of BMD compared with conventional stems.

## Methods

### Data collection and follow-up

#### Patients and methods

##### Trial design and participants

The study is a prospective, randomised controlled trial performed between October 2009 and August 2013 at the orthopaedic department of Danderyd Hospital, in collaboration with the Department of Clinical Sciences at Karolinska Institute, Stockholm, as described previously.^12^ The trial adhered to the Consolidated Standards of Reporting Trials (CONSORT) statement guidelines for proper reporting of randomised trials.^10^

We enrolled individuals diagnosed with primary osteoarthritis, scheduled for THA. Inclusion criteria comprised individuals aged 40–70 years with bone stock suitable for uncemented hip arthroplasty, femur type Dorr A or B according to Dorr et al.^13^ Exclusion criteria were: use of bisphosphonates, corticosteroids, cytostatic or other bone altering drugs in the last 6 months prior to surgery and BMI above 35 kg/m^2^.^12^

##### Implants

**Figure 1.**
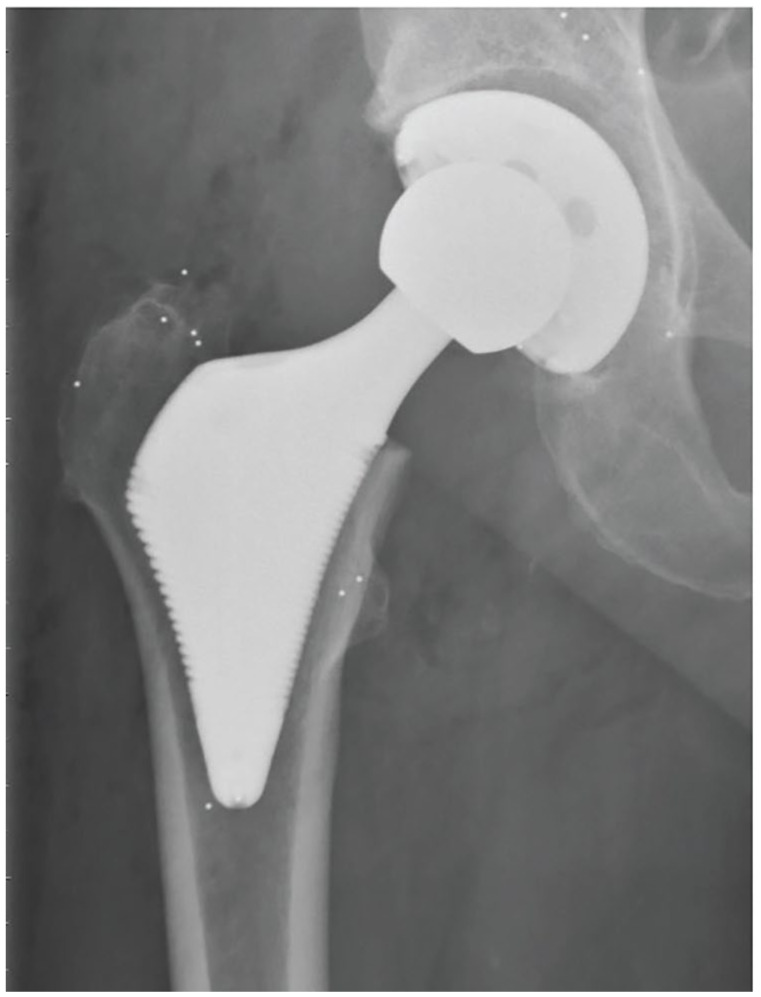
The ultra-short stem.

The treatment group received an ultra-short wedge-shaped porous and Hydroxyapatite (HA)-coated titanium stem (Proxima; Depuy Johnson and Johnson) (Figure 1), while the control group received a proximally porous and HA-coated, conventional tapered titanium stem (Bi-metric; Biomet) (Supplemental Table 1) Surgical design features of the ultra-short stem aimed for anatomical compatibility, featuring a wedge shape, lateral flare, and the absence of a diaphyseal stem. The stem’s surface macrotexture was stepped to enhance ingrowth and convert tangential forces into compressive loads on the bone.^14^

##### Surgery

Surgery was performed by 1 of 5 senior surgeons employing a posterolateral approach with posterior capsule and external rotator muscle repair.^15,16^ Hemispherical uncemented cups were used in both groups with 32-mm articulation and highly crosslinked polyethylene liners. Local infiltration analgesia was administered perioperatively, and full weight-bearing was permitted as tolerated. Surgeons were trained on the implant before using it clinically.^12^

##### Primary and secondary endpoints

**Figure 2.**
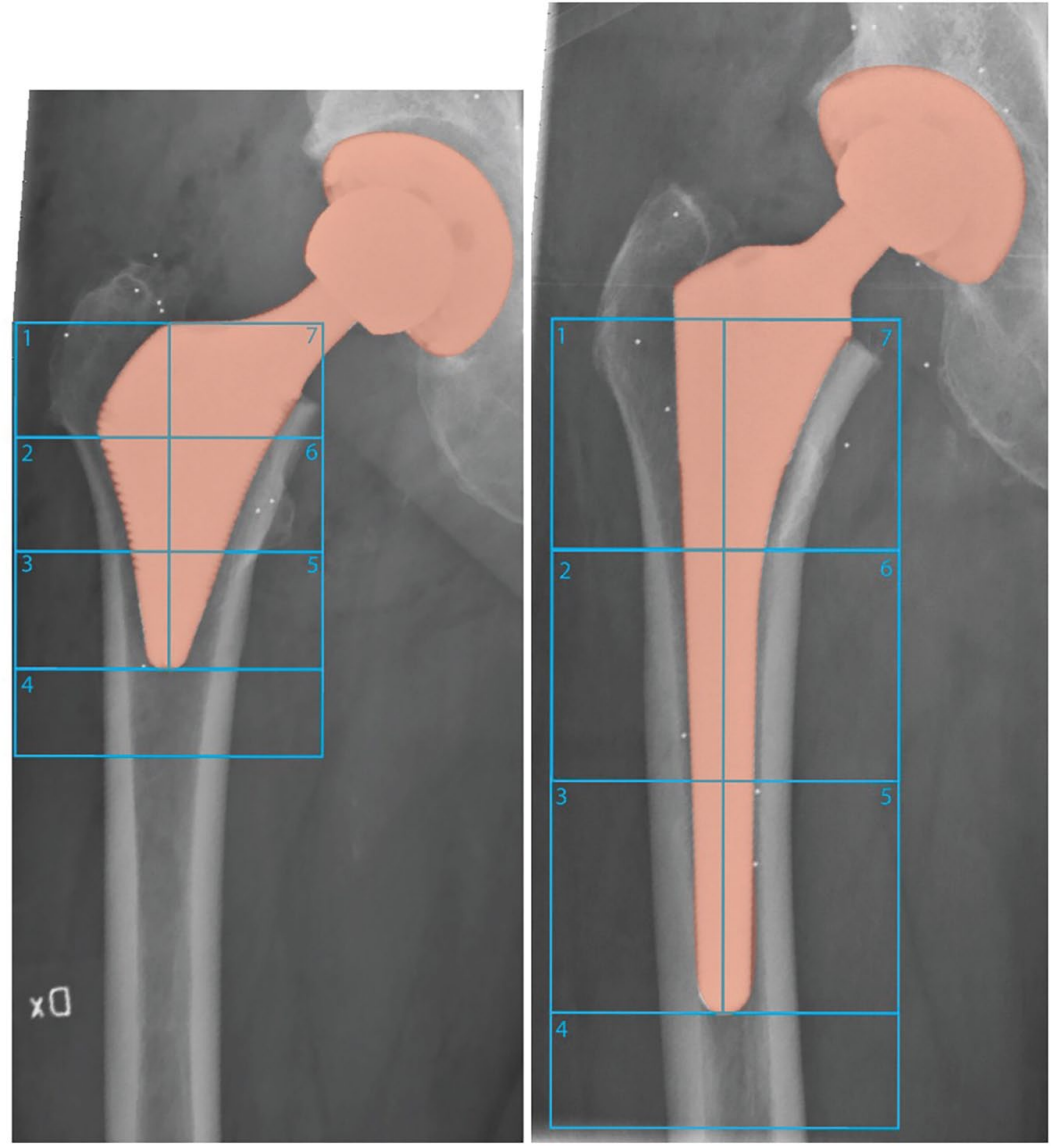
Gruen zones 1–7 measured from DEXA scanner.

The primary endpoint focused on changes in periprosthetic BMD in Gruen zones 1 and 7, measured at 6 and 10 years post-surgery. Gruen zones were adjusted to accommodate differences in stem lengths between the two stem types. Zones 1–2 and 6–7 in the ultra-short stem group were compared to zones 1 and 7, respectively, in the conventional stem group (Figure 2). Lumbar spine, L1–4, BMD measurements were used to estimate overall bone loss.

Secondary endpoints included alterations in periprosthetic BMD across all zones (Gruen zones 1–7) and functional clinical outcomes. Utilising a Lunar Prodigy Advance machine from General Electric Healthcare, dual-energy x-ray absorptiometry (DXA) scans measured changes in BMD at each zone, calculating the percentage ratio from baseline values obtained 2 days postoperatively.

##### Clinical outcome

Self-administered scores, Harris hip score (HHS) and EuroQol 5-dimensions (EQ-5D), were sent home to patients to assess clinical outcomes at each follow-up time point.

### Sample size and data analysis

Missing BMD data for patients with missing follow-up visits were addressed by employing the last observation forward method. Between-group comparisons of BMD were conducted using Student’s *t-*test, and a post-hoc Bonferroni correction, not originally planned, was applied to the primary endpoint to manage multiplicity. We assumed a difference in BMD of 15% in the proximal zones (Gruen 1 and 7) would be clinically relevant. A previous power analysis has shown that 15% difference in BMD at the 5% significance level would require 17 patients in each group and we assumed the same held true for our study.^17^ For the BMD analysis, we included only patients who had complete data for each follow-up time period. Clinical score data, which were non-normally distributed and ordinal, respectively, underwent analysis using Mann-Whitney U-test. All statistical analyses were performed using SPSS 22.0.

### Patient flow and baseline data

**Figure 3.**
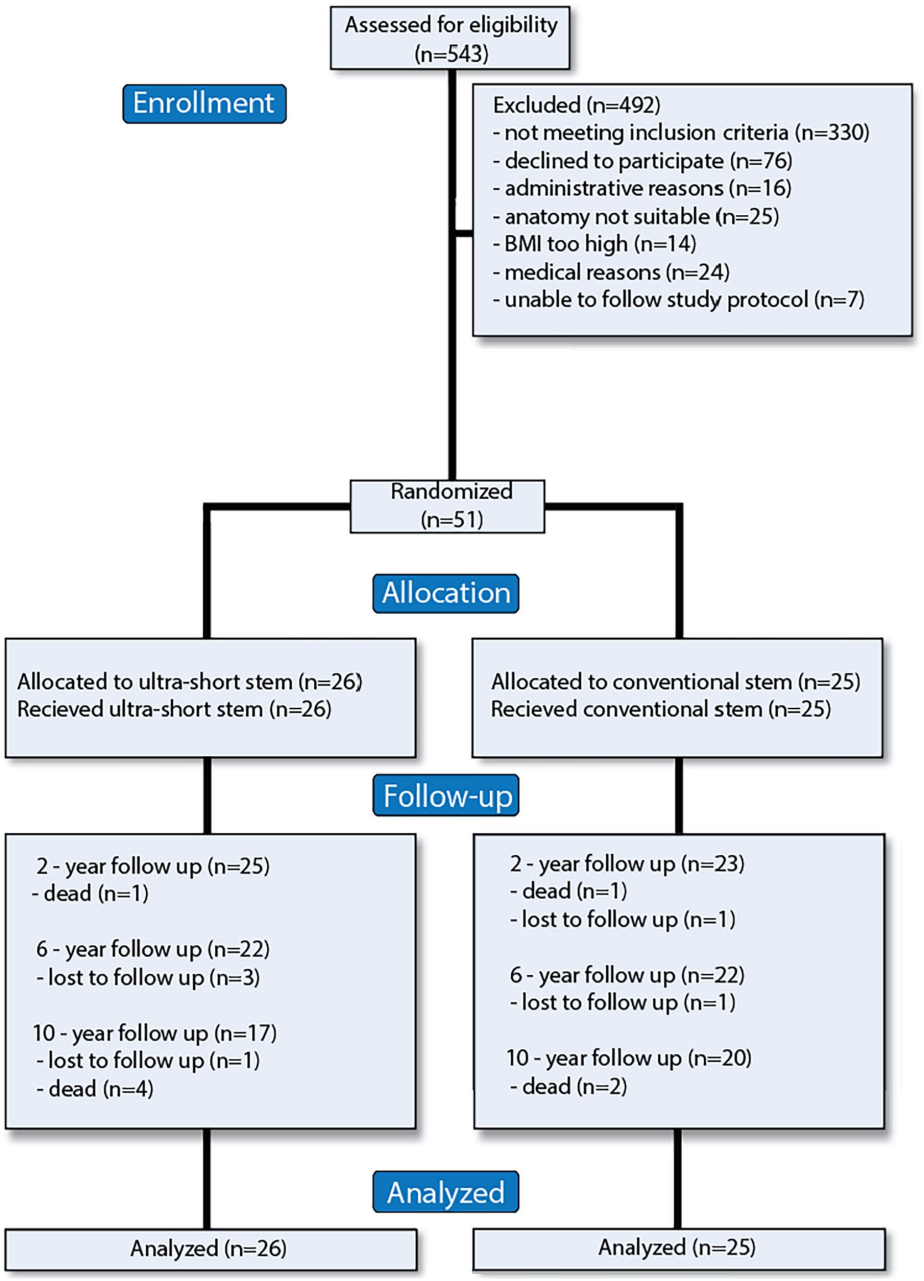
CONSORT flow diagram.

We enrolled 26 individuals in the ultra-short stem group, and 25 in the conventional stem group (Figure 3). The baseline characteristics of the 2 groups were similar (Table 1).

**Table 1.** Baseline characteristics of study participants.

Variable	Ultra-short	Control	*p*-value^b^
	*n* = 45^a^	*n* = 25^a^	
Sex			0.3
Female	21 (47%)	15 (60%)	
Male	24 (53%)	10 (40%)	
Age	61.0 ± 7.6	62.5 ± 5.9	0.5
Side			0.6
Right	22 (51%)	14 (58%)	
Left	21 (49%)	10 (42%)	
BMI	26.7 ± 4.0	27.5 ± 3.7	0.3
ASA			0.4
1	13 (32%)	11 (48%)	
2	21 (51%)	8 (35%)	
3	7 (17%)	4 (17%)	

BMI, body mass index; ASA, American Society of Anesthesiologists classification.

a *n* (%); mean ± standard deviation; ^b^Pearson’s chi-square test; Wilcoxon rank sum test; Fisher’s exact test.

### Ethics and registration

Local ethics committee approval (number 2008/4:3) and ClinicalTrials.gov registration (number NCT01319227) was obtained to ensure adherence to ethical standards and transparent trial documentation.

## Results

### Primary endpoints

**Figure 4.**
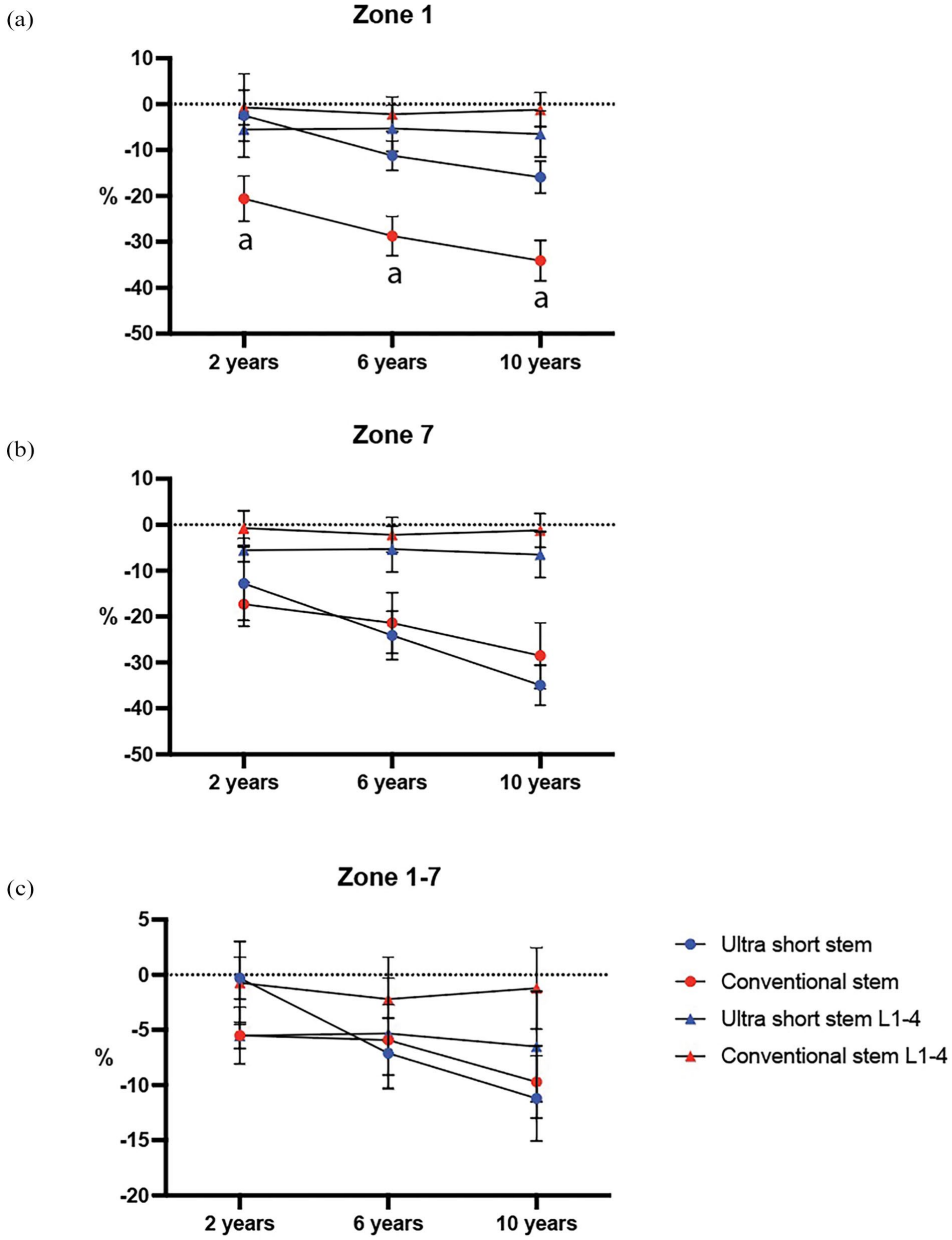
Mean percentage changed in BMD in Gruen zone 1 (a), 7 (b) and 1–7 (c). Error bars indicate 95% CI. Overall bone loss measured in L1-4 is indicated. Differences between stems were analysed using Student’s *t-*test. *p* < 0.05.

At 6 years and 10 years, the ultra-short stem group had a BMD difference of -17.6 (-23.4–-10.6) and -18.3 (-28.0–-9.0) respectively (*p*-values <0.001) in Zone 1. In Zone 7, at 6 years and 10 years, the reduction in BMD was similar between groups (Table 2). Both groups lost more BMD than their overall bone loss in both Zones 1 and 7 (Figure 4(a) and (b)). The conventional group lost significantly more BMD compared to the ultra-short group in Zone 1 relative to overall bone loss (Figure 4(a)) (Supplemental Table 2). There was no evidence of loosening of any of the stems during the follow-up period.

**Table 2.** Reoperations and adverse events.

Variable	Ultra-short	Control	*p*-value^b^
	*n* = 45^a^	*n* = 25^a^	
Reoperation			
Cup revision	1 (2.2%)	2 (8.0%)	0.3
DAIR	0 (0%)	0 (0%)	-
Stem revision	4 (8.9%)	1 (4.0%)	0.6
Closed reduction	1 (2.2%)	1 (4.0%)	>0.9
Complications			
Any	5 (11%)	3 (12%)	>0.9
Dislocation	1 (2.2%)	1 (4.0%)	>0.9
PJI	0 (0%)	1 (4.0%)	0.4
Non union	0 (0%)	0 (0%)	-
Vancouver C	0 (0%)	0 (0%)	-
Vancouver B1	1 (2.2%)	0 (0%)	>0.9
Vancouver B2	0 (0%)	0 (0%)	-
Stem loosening	3 (6.7%)	0 (0%)	0.5
Cup loosening	0 (0%)	1 (4.0%)	0.4

DAIR, debridement, antibiotics and implant retention.

a *n* (%); Mean ± standard deviation; ^b^Pearson’s chi-square test; Wilcoxon rank sum test; Fisher’s exact test.

### Secondary endpoints

There were no significant differences between BMD 1–7 between groups at 6 and 10 years post-surgery (Table 1) (Figure 4(c)).

At the 6-year follow-up, both stem groups exhibited improvements in HHS from baseline. In the ultra-short stem group, the HHS improved to a mean of 91, while the conventional stem group showed an improvement to a mean of 88, the differences between groups were not significant. The ultra-short stem group demonstrated a mean of 84 in EQ-5D score, whereas the conventional stem group reported a mean of 79. Upon reaching the 10-year follow-up, both stem groups maintained improved HHS from baseline, although scores slightly decreased compared to the 6-year follow-up. The ultra-short stem group showed a mean HHS of 91, whereas the conventional stem group exhibited a mean HHS of 86. The EQ-5D scores at the 10-year mark for both groups were also relatively stable, with the ultra-short stem group reporting a mean EQ-5D score of 79, and the conventional stem group reporting a mean EQ-5D score of 79 (Table 3).

**Table 3.** Clinical outcome scores and 6 and 10 years follow-up.

Time point	Variable	Ultra-short stem (*n* = 26)	Conventional stem (*n* = 25)
**Baseline**	Age, years^a^	65 (5)	62 (6)
Male/ Female, *n*		11/15	11 /14
Harris Hip Score, pre-op ^b^		56 (29–68)	46 (10–70)
	EQ-5D	69 (64–74)	69 (66–72)
**6-year follow-up**			
	Male	11	11
	Female	11	11
	Harris Hip Score	91 (60–100)	88 (49–100)
	EQ-5D	84 (65–100)	79 (50–100)
**10-year follow-up**			
	Male	9	10
	Female	8	10
	Harris Hip Score	91 (63–100)	86 (51–100)
	EQ-5D	79 (40–100)	79 (48–100)

a mean (standard deviation); ^b^median (range).

### Adverse events

8 of 51 patients sustained an adverse event. Ultra-short stems had a few more mechanical complications compared to conventional stems. The ultra-short stem group had 1 dislocation, 1 Vancouver C fracture and 1 aseptic stem loosening. The conventional group had 2 cases of prosthetic joint infection and 1 dislocation.

In the conventional group, 1 case underwent debridement, antibiotics, and implant retention (DAIR), while in the ultra-short stem group, internal fixation (ORIF) was performed in 1 case. The total number of reoperations was identical between the 2 groups, with 2 cases each (Table 4).

**Table 4.** Adverse events and reoperations during the follow-up period.

Adverse events	Ultra-short stem (*n* = 26)	Conventional stem (*n* = 25)
Dislocation	1	1
Prosthesis joint infection		2
New PFX distal to the stem (Vancouver C)	1	
Loosening of stem	1	
Back pain	1	
Trochanteritis		1
Reoperations		
DAIR		1
Stem revision	1	1
ORIF	1	
Total reoperation (*n*)	2	2
Total complications (*n*)	6	6

PFX, periprosthetic fracture; ORIF, open reduction and internal fixation; DAIR, debridement, antibiotics and implant retention.

## Discussion

In this long-term follow-up study, we found significant differences in BMD changes between the ultra-short stem and conventional stem groups in zones proximal to the femoral stem.

In summary, the short stems had less bone loss in zone 1, but no statistically significant difference in zone 7 and when comparing all zones. The ultra-short stem group also lost less BMD relative to overall bone loss compared to the conventional stem group. The groups exhibited similar HHS and corresponding adverse events. Based on this, it appears that short stems may be preferable in order to preserve bone density in the proximal region around the stem. These findings are in line with prior observations at 24 months post-surgery, indicating that ultra-short stems might have an advantage in mitigating periprosthetic bone loss.^12^

This is, to our knowledge, the first long-term follow-up study of a randomised controlled trial of ultra-short versus conventional stems in THA. Previous studies measuring BMD changes between short and conventional stems in THA have also found that short stems preserve BMD in the proximal regions, although not to the extent shown in our long-term follow-up.^18^ Despite preserving BMD, short stems are prone to mechanical complications. This is not a new finding and should be considered by surgeons when choosing patients.

The earlier observation of reduced BMD loss in the calcar region with the ultra-short stem, although not consistently significant beyond 2 years post-surgery, might be attributed to load distribution from the stem’s medial contour and preserved calcar bone following high neck resection. This preservation of femoral bone density underscores the importance of stem length in minimising femoral bone shielding. Our findings re-affirm our previous findings regarding the ultra-short stem’s potential to mitigate periprosthetic bone loss, in Zone 1, compared to the conventional stem. The preservation of periprosthetic bone density in the proximal femoral regions is an important factor in reducing the risk of stress shielding and late periprosthetic fractures.

Short femoral stems have gained in popularity due to excellent clinical and radiographic mid-terms results; however, most studies have not been able to show that short stems reduce BMD loss in the proximal femoral regions, making our findings novel and welcome.^19–23^ 1 observation worth mentioning is that zone 7 was similar in both stems. Bone loss in zone 7 is commonly reported in THA and a recent study has found that stem morphology is important in bone preservation, in particular in zone 7.^24^

Clinical assessments through HHS and EQ-5D revealed favourable outcomes for both stem groups at 6 and 10 years post-surgery which is in line with previous studies.^25–27^ The ultra-short stem group exhibited high HHS and EQ-5D scores, indicating improved hip functionality and overall quality of life compared to baseline. It is worth noting that our patient cohort possessed adequate bone stock and that compromised bone stock could potentially impact clinical outcomes in THA procedures. While some studies have reported favorable outcomes utilising the same stem design, further investigation is necessary to ascertain its performance in scenarios involving compromised bone stock which might influence results.^28–30^

The incidence of adverse events and complications, including dislocation, joint infection, periprosthetic fracture, subsidence/loosening, and reoperations, was similar between the ultra-short and conventional stem groups. While both stem groups encountered comparable adverse events, the occurrences were relatively low. The groups experienced different adverse events, although no statistical difference was detected. There were 2 reoperations in the ultra-short stem group due to aseptic loosening or fracture while the conventional group had no re-operations due to fracture or loosening. Whether the ultra-short stem predisposes patients to these complications cannot be determined within the scope of our study but such complications could be due to inherent factors associated with the ultra-short stem such different surgical techniques, larger metaphyseal area and the need for intramedullary adjustment. We did not find any difference in mortality or complications, most likely due to our small sample size, although other studies have reported a higher incidence of complications using conventional stems.^10,31,32^

This study has certain limitations, including a relatively small sample size and a single-centre setting, which might limit the generalisability of the findings. The lack of patient blinding and a potentially underpowered sample size due to post hoc statistical adjustments also warrant cautious interpretation. Lastly, we compare zone 1 and zone 2 in the ultrashort stem group with zone 1 in the conventional stem group. While these zones are similar, they do not correspond exactly to the same anatomical area. The strengths of our study include a prospective randomised design with high follow-up rates and sensitive evaluation methods, adhering to the intention-to-treat principle.

Although our results suggest that ultra-short stems can preserve bone density and reduce stress shielding, a holistic view, including complications and implant survival, must be considered when evaluating the clinical utility of these stems. It is important to acknowledge that BMD reduction alone may not provide a complete picture of the stem’s clinical performance. The potential for higher rates of complications, such as loosening or periprosthetic fractures, particularly with ultra-short stems, must be carefully considered. Radiological assessments like subsidence, proximal femoral bone shape and canal filling were not part of our study but are important for understanding the overall mechanical performance of the stem. Future studies with more comprehensive radiological follow-up and larger sample sizes are needed to confirm the long-term clinical benefits and risks associated with ultra-short stems.

## Conclusion

The use of ultra-short stems in THA can preserve periprosthetic bone density up to 10 years postoperatively and can potentially enhance long-term clinical outcomes without increasing the risk of adverse events. Patient selection is likely a crucial contributor to successful outcomes. Further studies are warranted in order to elucidate the full potential of ultra-short stems in THA.

## Supplemental Material

sj-pdf-1-hpi-10.1177_11207000251371283 – Supplemental material for Long-term evaluation of periprosthetic bone changes in ultra-short versus conventional stems in total hip arthroplasty: a 10-year follow-up of a randomised controlled trial Supplemental material, sj-pdf-1-hpi-10.1177_11207000251371283 for Long-term evaluation of periprosthetic bone changes in ultra-short versus conventional stems in total hip arthroplasty: a 10-year follow-up of a randomised controlled trial by Michael Axenhus, Mats Salemyr, Sebastian Mukka, Martin Magnèli and Olof Sköldenberg in HIP International

sj-pdf-2-hpi-10.1177_11207000251371283 – Supplemental material for Long-term evaluation of periprosthetic bone changes in ultra-short versus conventional stems in total hip arthroplasty: a 10-year follow-up of a randomised controlled trial Supplemental material, sj-pdf-2-hpi-10.1177_11207000251371283 for Long-term evaluation of periprosthetic bone changes in ultra-short versus conventional stems in total hip arthroplasty: a 10-year follow-up of a randomised controlled trial by Michael Axenhus, Mats Salemyr, Sebastian Mukka, Martin Magnèli and Olof Sköldenberg in HIP International
