# Supplementary material for: Long-term evaluation of periprosthetic bone changes in ultra-short versus conventional stems in total hip arthroplasty: a 10-year follow-up of a randomised controlled trial
Source: Hip Int. 2025 Dec 18;36(1):55–63. doi: 10.1177/11207000251371283 (PMC12876410; doi:10.1177/11207000251371283)
Supplement: sj-pdf-1-hpi-10.1177_11207000251371283 – Supplemental material for Long-term evaluation of periprosthetic bone changes in ultra-short versus conventional stems in total hip arthroplasty: a 10-year follow-up of a randomised controlled trial [file sj-pdf-1-hpi-10.1177_11207000251371283.pdf]

Stem propertie
Design
Material
Coating
Stem length

Ultra-short stem	Conventional stem
Anatomically wedge-shaped	Straight tapered
Titanium alloy (Ti-6AI-4V)	Titanium alloy (Ti-6AI-4V)
Fully porous-coated with sintered beads. Covered with hydroxyapatite. Distal tip textured	Proximal porous-coated, covered with plasma-sprayed hydroxyaptite.
71-83 mm	130-155 mm
