# Supplementary material for: Long-term evaluation of periprosthetic bone changes in ultra-short versus conventional stems in total hip arthroplasty: a 10-year follow-up of a randomised controlled trial
Source: Hip Int. 2025 Dec 18;36(1):55–63. doi: 10.1177/11207000251371283 (PMC12876410; doi:10.1177/11207000251371283)
Supplement: sj-pdf-2-hpi-10.1177_11207000251371283 – Supplemental material for Long-term evaluation of periprosthetic bone changes in ultra-short versus conventional stems in total hip arthroplasty: a 10-year follow-up of a randomised controlled trial [file sj-pdf-2-hpi-10.1177_11207000251371283.pdf]

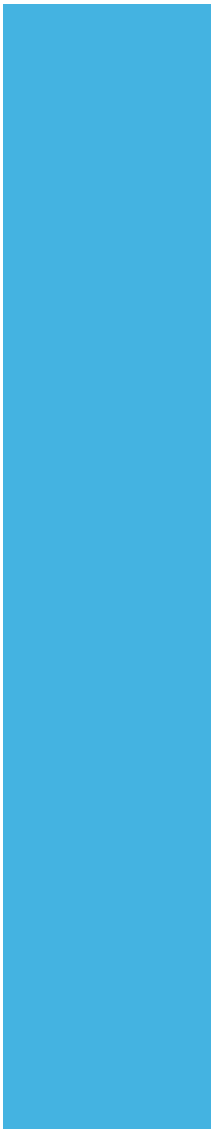

			BMD (SD) Ultra short stem (n=26)	BMD (SD) Conventional stem (n=25)
**Zone 1**				
	Preop	(n = 16 / 17)	0.9852 (0.27)	1.216 (0.14)
	6 years	(n = 16 / 17)	0.8773 (0.26)	0.8902 (0.33)
	10 years	(n = 16 / 17)	0.826 (0.31)	0.833 (0.18)
**Zone 7**				
	Preop	(n = 16 / 17)	1.426 (0.16)	1.573 (0.29)
	6 years	(n = 16 / 17)	1.083 (0.28)	1.237 (0.26)
	10 years	(n = 16 / 17)	0.963 (0.32)	1.126 (0.35)
**Zone 4**				
	Preop	(n = 16 / 17)	2.053 (0.22)	1.917 (0.23)
	6 years	(n = 16 / 17)	2.013 (0.24)	1.854 (0.28)
	10 years	(n = 16 / 17)	1.964 (0.29)	1.813 (0.26)
**Zone 1-7**				
	Preop	(n = 16 / 17)	1.443 (0.21)	1.916 (0.26)
	6 years	(n = 16 / 17)	1.34 (0.26)	1.794 (0.22)
	10 years	(n = 16 / 17)	1.28 (0.26)	1.719 (0.31)
**L1-4**				
	Preop	(n = 16 / 17)	1.208 (0.19)	1.19(0.19)
	6 years	(n = 16 / 17)	1.144 (0.21)	1.169 (0.21)
	10 years	(n = 16 / 17)	1.131 (0.21)	1.18 (0.19)

p-value determined with student's t-test
Last observation carried forward was used twice due to missing data.

BMD loss relative to overall bone loss (%) (95% CI)		overall bone loss (%) (95% CI)	
Ultra short stem (n=26)	p-values	Conventional stem (n=25)	p-values
-5.7 (-14.1 to 2.7)	0.128	-25.0 (-32.8 to -17.2)	<0.001
-9.8 (-16.7 to -2.9)	0.042	-30.7 (-42.1 to -19.3)	<0.001
-18.8 (-27.9 to -9.7)	<0.001	-19.6 (-30.1 to -9.1)	<0.001
-26.1 (-37.3 to -14.9)	<0.001	-27.6 (-41.1 to -14.1)	<0.001
-1.6 (-5.2 to 2.1)	0.173	-4.6 (-10.1 to 0.9)	0.279
-2.2 (-6.4 to 2.2)	0.115	-7.2 (-16.4 to -4.0)	0.082
-1.8 (-16.6 to 13.0)	0.204	-4.6 (-16.9 to 7.1)	0.231
-4.9 (-21.2 to 11.4)	0.322	-9.4 (-22.4 to 3.6)	0.142
